# Revisiting the Optical Spectrum of the Plutonyl Ion (PuO_2_)^2+^ in 1 M HClO_4_

**DOI:** 10.1021/acs.jpca.4c05837

**Published:** 2024-12-18

**Authors:** Norman M. Edelstein

**Affiliations:** MS 70A3317, Chemical Sciences Division, Lawrence Berkeley National Laboratory, Berkeley, California 94720, United States

## Abstract

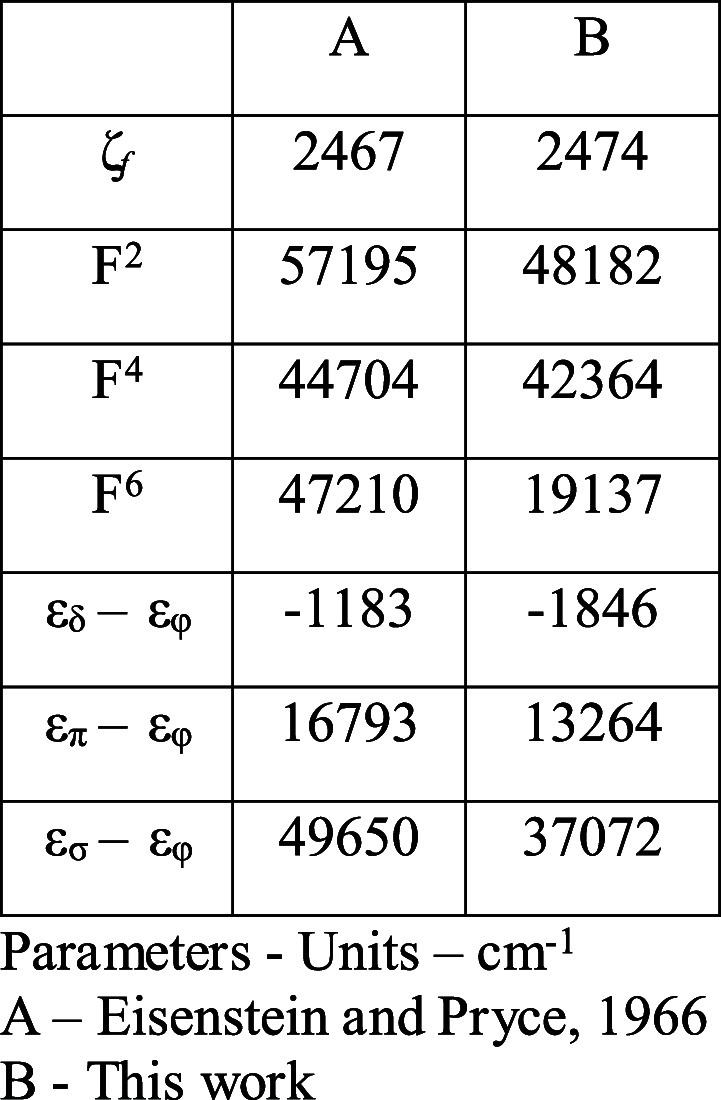

The analysis of the solution absorption spectrum of the plutonyl ion in an aqueous environment was given by Eisenstein and Pryce (E&P) in 1968. In 2011 a new spectrum was published of the (PuO_2_)^2+^ ion in 1 M HClO_4_. We have been provided with the original data of this spectrum and have found in the data a previously unreported low-lying transition at 7385 cm^–1^ which we have assigned as a magnetic dipole transition. We have fit most of the near-infrared and optical transitions with Gaussian fits and tabulated a new energy level list up to 22,000 cm^–1^ which mostly agrees with the data of E&P. We assumed a crystal field of D_∞h_ (only axial symmetry) and utilized the intensity calculations published for the isoelectronic (NpO_2_)^1+^ ion using a complete basis set for the 5f^2^ problem including the Coulombic, spin–orbit as well as the crystal field Hamiltonian. Our results differ substantially from those of E&P. Subsequently, we used a truncated Hamiltonian to try to establish the effects of assuming the σ antibonding orbitals are at such high energies that we can ignore their contributions to the lower lying φ and δ orbitals.

## Introduction

The first recorded optical spectra of the plutonyl ion, (PuO_2_)^2+^, (consisting of two 5f electrons added to the closed shell of the UO_2_^2+^ ion), in aqueous solution were reported during the 1940s and shortly afterward.^1−3^ Electron paramagnetic resonance (EPR) experiments followed shortly on the plutonyl ion diluted into single crystals of uranyl salts, establishing the major component of the ground term of the plutonyl ion as ^3^H_4_.^4,5^ Eisenstein and Pryce, in a series of papers culminating in the 1960s,^6−9^ developed the theory for the interpretation of the magnetic and optical data for the actinyl ions, NpO_2_^2+^, NpO_2_^1+^, and PuO_2_^2+^ in aqueous solution. Subsequently, Denning and co-workers published detailed experimental data and theoretical interpretation of high resolution optical studies on single crystals of Cs_2_UO_2_Cl_4_ and CsUO_2_(NO_3_)_3_ and showed the optical spectrum in these crystals are charge transfer transitions that begin at energies greater than 20,000 cm^–1^.^10−12^ These uranyl crystals also have proved suitable for diluting heavier actinide ions such as NpO_2_^2+^ and PuO_2_^2+^ in order to study their optical and magnetic properties below 20,000 cm^–1^. Denning, et al. have grown doped crystals of Cs_2_NpO_2_Cl_4_ diluted in Cs_2_UO_2_Cl_4_^13^ and Gorshkov and Mashirov have conducted optical studies of Cs_2_PuO_2_Cl_4_ diluted in Cs_2_UO_2_Cl_4_.^14,15^ More recently, fluorescence has been observed from the NpO_2_^2+^ ion and the PuO_2_^2+^ ion diluted into crystals of Cs_2_UO_2_Cl_4_.^16,17^ The detailed studies of Denning, et al. have provided important information which, along with the theoretical calculations of Matsika, et al.^18^ of the intensities of NpO_2_^1+^ ion in aqueous solution (isoelectronic with the (PuO_2_)^2+^ ion), which we utilized to reanalyze the optical spectral features of the aqueous neptunyl ion.^19^

A number of theoretical papers have discussed the electronic structure of actinyl ions including the plutonyl ion in aqueous solution.^18,100−23^ In order to compare their results with the experimental data on the plutonyl ion, they compared their theoretical results with the energy level analysis given by Eisenstein and Pryce in 1968. Much later the optical spectrum of the plutonyl ion in 1 M HClO_4_ was reported by the LANL group in 2012^24^ in the energy range of 8000 to 12,000 cm^–1^ in their supplementary material. In fact, their data covered the energy range of 5000 to 25,000 cm^–1^. The original data has kindly been sent to us and we have remeasured and analyzed the optical spectrum of the plutonyl ion in the energy range from 7300 to 22,000 cm^–1^. Energies below ∼7300 cm^–1^ in the spectrum are dominated by the overtone and combination frequencies from the stretching and deformation of the O–H groups of the solvent H_2_O.^25^ In this paper we reassign the plutonyl spectrum in perchloric acid and refit the Hamiltonian parameters, based primarily on the intensities calculated by Matsika et al.^18^ and our earlier studies for the NpO_2_^1+^ ion.^19^

## Review of Earlier Work

**Figure 1:**
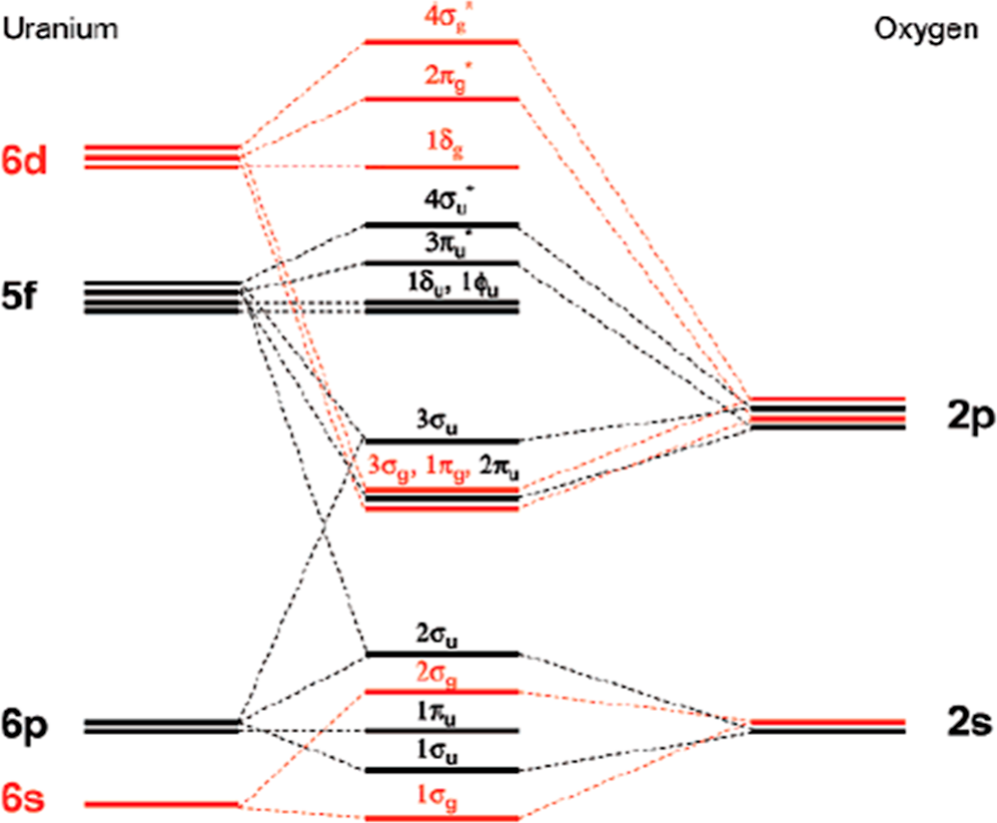
Schematic energy level diagram for actinyl valence orbitals. The red colored levels represent gerada (g) or symmetric orbitals, the black colored levels represent ungerada (u) or antisymmetric orbitals. Reprinted with permission from Denning, R. G., “Electronic Structure and Bonding in Actinyl Ions and their Analogs,” J. Phys. Chem. A 2007, 111, 4125–4143, Copyright 2007 American Chemical Society.

The results of earlier spectroscopic studies on the uranyl and neptunyl ions have been summarized by Denning in two review articles.^26,27^ The earliest review summarized the experimental details of the spectroscopic studies up to the past decade of the twentieth century. The later review included X-ray spectroscopic studies of the uranyl ion and theoretical calculations on its electronic structure. For the present study, we utilize the energy level diagram that Denning has given for the uranyl ion and it is shown in Figure 1.

For our purposes this figure shows the 5f orbitals, 1δ_u_ and 1φ_u_ as nonbonding under D_∞h_ symmetry. The 3π_u_ and 4σ_u_ orbitals participate in bonding and these antibonding 5f orbitals are shifted to higher energies. Thus, the two 5f electrons for the plutonyl ion (PuO_2_)^2+^ in aqueous solution will begin to fill the two nonbonding δ_u_ and φ_u_ orbitals, at least for the lowest energy orbitals. At higher energies, significant contributions from the 3π_u_ and 4σ_u_ orbitals will begin to appear. In this paper we utilize conventional 5f electron crystal field theory for an analysis of the levels up to approximately 22,000 cm^–1^. From this point on in this work, we refer to the 5f electrons discussed above as δ_u_ and φ_u_ for 1δ_u_ and 1φ_u_ nonbonding orbitals and π_u_ and σ_u_ for 3π_u_* and 4σ_u_* antibonding orbitals.

We also discuss probable errors arising from the use of conventional f-electron crystal field theory in cases where it is clear that the antibonding π_u_ and σ_u_ should be treated differently than the 1δ_u_ and 1φ_u_ nonbonding orbitals.^28^ We do so by using a limited basis set which drops all matrix elements that contain the lz = 0 components (σ_u_ components) and then redo the crystal field fitting of the observed low-lying energy levels consisting of levels primarily composed of only δ_u_ and φ_u_ components but also with some π_u_ components.

## Review of Theory

The phenomenological theory utilized in this paper has been summarized in our earlier analysis on the aqueous (NpO_2_)^+^ ion.^19^ We briefly summarize the theory here.

For an f^n^ ion, the observed energy levels can be fitted to a phenomenological Hamiltonian *H* = *H*_FI_ + *H*_CF_ by a simultaneous diagonalization of the free-ion Hamiltonian *H*_FI_ and the crystal field Hamiltonian *H*_CF_. The free-ion Hamiltonian is given as follows
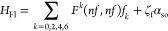
1where *F*^*k*^*(nf,nf)*’s and ζ_f_ represent the radial parts of the electrostatic and spin–orbit interaction, respectively, between f electrons, and *f*_*k*_ and α_so_ are the angular parts of these interactions. For the different interactions the angular parts can be evaluated exactly; the radial parts, *F*^*k*^ and ζ_f_, the Slater and spin–orbit coupling parameters respectively, are evaluated empirically. Since the parameter F^0^ shifts all levels of the f^*n*^ ion equally, and we are interested only in the relative energies of the ground configuration of the plutonyl ion, we need to fit only the free ion parameters, F^2^, F^4^, F^6^ and ζ_f_.

The crystal field Hamiltonian is expressed in terms of the phenomenological crystal field parameters *B*_*q*_^*k*^ which are defined in Wybourne^29^ and the angular tensor operators *C*_*q*_^*k*^ as follows
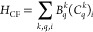
2where the sum involving *i* is over all the f electrons. The values of *k* and *q* are limited by the point symmetry of the f^*n*^ ion site. For states of the same parity, k will have only even values. The term for which *k* = 0 and *q* = 0 shifts all levels of an f^n^ configuration equally and is not utilized in fitting levels within one configuration.^29^ For the actinyl ions it is convenient to define the crystal field Hamiltonian as
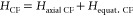
3with the axial crystal field defined as
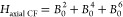
4

EXAFS data indicate that in aqueous solution the coordination sphere about the equatorial axis for actinyl ions consist of five water molecules bonded to the actinyl moiety through the O atoms.^30^ Matsika et al. developed a five-coordinate equatorial crystal field model to calculate theoretical transition intensities for the (NpO_2_)^+^ molecule which is isoelectronic with the plutonyl ion.^18^ Since the ground level for both the (NpO_2_)^+^ and the (PuO_2_)^2+^ ions are similar we utilize this model and assume the relative intensities for the plutonyl levels with approximately the same composition as the neptunyl states will be similar although their actual energies will be shifted.

E&P in their analyses of the 5f^1^ and 5f^2^ actinyl compounds utilized a different basis set for the crystal field Hamiltonian. They defined the crystal field as the differences in the energies of the 5f orbitals in a very strong axial crystalline field as found for uranyl type compounds. In a strong axial crystal field a single 4f or 5f orbital is split into four energy levels defined by the orbital angular momentum quantum number lz where lz = 0 or 5fσ, ± 1or 5fπ, ±2 or 5fδ, ±3 or 5fφ. Each of these orbitals has a spin quantum number associated with it, either sz = ± 1/2. Thus, for the f orbitals we have 7 orbital states and a total (with spin) of 14 states. E&P chose to set the level 5fφ = 0.0 cm^–1^ and with this definition we have three crystal field parameters defined as 5fδ–5fφ, 5fπ–5fφ, and 5fσ–5fφ which we refer to in this paper as the δ, π, and σ parameters. The relationships between the *B*_0_^*k*^ as defined by Wybourne^29^ and the crystal field parameters utilized by E&P and the Denning group are shown in Table 1.

**Table 1:** Definition of the Orbitals φ, δ, π, σ in Terms of *B*_0_^2^, *B*_0_^4^, *B*_0_^6^ (Wybourne Notation^29^) from Abragam and Pryce,^31^ Appendix B, Tables 17–18^a^


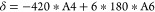
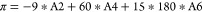
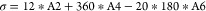
with A2 = α∗(*B*_0_^2^/2), A4 = β∗(*B*_0_^4^/8), A6 = γ∗(*B*_0_^6^/16)
and α = –2/45, β = 2/(11∗45), γ = −4/(11∗13∗27)

a In order to obtain three crystal field parameters to fit the f^1^ energy levels, E&P defined the following three parameters, δ – φ, π – φ – φ, σ – φ, that is setting the energy of the φ orbital to 0 cm^−1^. In this paper we sometimes refer to the orbitals, δ – φ, π – φ – φ, σ – φ as δ, π, and σ.

The actinyl crystal field is very strong and has been evaluated by Denning et al.^13^ in their studies of NpO_2_^2+^ ion diluted in single crystals of Cs_2_UO_2_Cl_4_ and CsUO_2_(NO_3_)_3_. The *B*_*q*_^*k*^ parameters (*q* > 0) are appreciably smaller than the *B*_0_^*k*^ parameters in these crystals. We anticipate the equatorial crystal field for the aqueous species to be considerably weaker than found in the above crystalline samples. In our calculations we assume D_∞h_ symmetry for the 5f^2^ configuration and use the group theory notation given in Table 2.

**Table 2:** Group Theory Labeling of States for a Crystal Field of D_∞h_. The corresponding values for jz in the (sz1,lz1,sz2,lz2) basis set and Jz in the SLJJz representations are given.

D_∞h_^a^	D_∞h_ j_z_ value	D_∞h_ Jz value
A_1g_ (Σ_*g*_^+^)	0	0
A_2g_ (Σ_*g*_^–^)	0	0
E_1g_ (π_g_)	±1	±1
E_2g_ (Δ_g_)	±2	±2
E_3g_ (φ_g_)	±3	±3
E_4g_	±4	±4
E_5g_	±5	±5
E_6g_	±6	±6

a Ref. (32).

E&P^8,9^ carried over the use of this CF basis set to their studies of the axial 5f^2^ ions, (NpO_2_)^1+^ and the (PuO_2_)^2+^ ions. We call this basis set the (sz1,lz1,sz2,lz2) basis set and the angular factors for the *F*^*k*^*s* defined above can be evaluated using the tables from Condon and Shortley.^200^ Since the spin–orbit and crystal field Hamiltonians are one-electron operators, their matrix elements can be evaluated by properly summing the one electron energies for the each of the electrons in the (sz1,lz1,sz2,lz2) basis set. The F^*k*^ radial parameters are defined the same in both basis sets. It is straightforward to define the crystal field energy levels in (sz1,lz1,sz2,lz2) basis set in terms of the *B*_0_^*k*^ parameters and vice versa using the equations given in Table 1 as obtained from the tables of Abragam and Bleaney.^31^ In the work described below we have used both basis sets for our calculations.

We found in the analysis of NpO_2_^+^ ion in aqueous solution assuming D_5h_ symmetry, that there was no observed structure for the states that should be split by this equatorial crystal field.^19^ In Table 3 we show the energy levels for the plutonyl aqueous ion calculated with the parameters of E&P^9^ with and without the equatorial crystal field parameters.

**Table 3:** Comparison of the Calculated Energy Levels with the Parameters Obtained by E&P^9^ with and without the Inclusion of the E&P Term V_6_ (which is Equivalent to *B*_6_^6^ in the Wybourne convention)^a^

energy levels D_∞h_	calc. energies D_∞h_ (cm^–1^)	E&P calc. energies with V_6_
E_4g_	0	0
A_1g_	2444	2445
E_1g_	4259	4258
E_5g_	7130	7133
A_2g_	10165	10157
E_1g_	10486	10489
A_1g_	10645	10640
E_2g_	11889	11892
E_6g_^b^	12856	12862, 12866
E_4g_	15467	15469
A_1g_	15979	15980
E_1g_	17875	17877
E_3g_^b^	19083	19080, 19086
A_1g_	19779	19780
E_2g_	21251	21253
E_5g_	21890	21885

a The E&P parameters are converted into the Wybourne convention (except for V_6_), all units in cm^–1^ are as follows: F^2^ = 57195, F^4^ = 44703.5, F^6^ = 47210.2, ζ = 2467, *B*_0_^2^ = 53456.8, *B*_0_^4^ = 74581.7, *B*_0_^6^ = 44108.4, V_6_ = 168.

b These doubly levels degenerate levels will split with the addition of the V_6_ term.

As can be seen from this table, the inclusion of the equatorial crystal field parameters results in a splitting of a few cm^–1^ which is much less than the line widths and errors in the measurements. Therefore, we shall ignore the equatorial crystal field parameters and use only the axial parameters in this work. A more serious problem with the analysis of E&P that in their analysis F^6^ is greater than F^4^ as shown in Table 3 because, as stated by Condon and Shortley, “F^k^ is essentially positive and a decreasing function of k.”^33^

Most calculations given in this paper have included all 91 states of the f^2^ configuration. In this case we have a complete basis set so it makes no difference whether we use the SLJJz basis set or the (sz1,lz1,sz2,lz2) basis set, the numerical results will be the same as long as we use the same effective parameters for the calculations. However, we note that we are assuming that conventional Hamiltonian for f^2^ electrons as described above can be utilized for the plutonyl ion even though, as we described above, it should only be applicable for the two nonbonding δ_u_ and φ_u_ orbitals.^28^ This is the same approximation that E&P made and that we utilized in our earlier study of the neptunyl ion. Later in this work we will test this assumption by using a reduced basis set without σ orbitals to see what effect this has on the parameter values.

### Analysis of Experimental Results

We utilize the experimental data for (PuO_2_)^2+^ ion dissolved in 1 M HClO_4_ published by Gaunt et al.^24^ In their paper Gaunt et al. reported the optical spectrum of 1.52 mM Pu(VI) in 1.0 M HClO_4_ at room temperature (supplementary information, Figure S2, bottom graph) in the wavelength range from 360 to 1200 nm (8333–27,778 cm^–1^). In fact, data was taken from 1500 to 360 nm with a fixed spectral bandwidth of 0.2 nm (Dr. Sean Reilly, private communication). This Excel data file was kindly sent to us by Dr. Sean Reilly of LANL.

**Figure 2:**
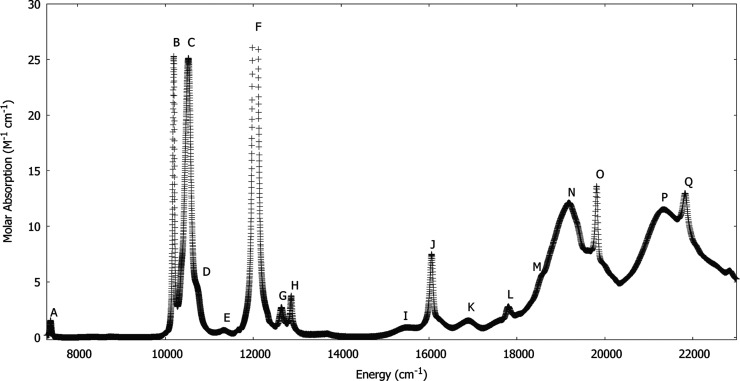
Absorption spectrum at room temperature of the [PuO_2_]^2+^ ion in aqueous 1 M HClO_4_.

Upon further examination of this data, we found a weak, sharp feature at 7385 cm^–1^ (1354 nm) which we assign to a weak magnetic dipole transition analogous to the feature assigned as a weak magnetic dipole transition at 6173 cm^–1^ in the (NpO_2_)^1+^ spectrum. At energies lower than 7300 cm^–1^ the spectra are dominated by features attributed to the solvent, aqueous 1 M HClO_4_. The experimental data at room temperature are shown in Figure 2.

The experimental spectrum was divided into regions and the bands in each of these regions were fit with a series of Gaussian line shapes. Depending on the region, in some cases the background was included as a constant or another line to be fit. The results of this fitting procedure are given in Table 4.

**Table 4:** Measured Peaks (*x*), Half Widths (*s*), and Areas (*A*) from Gaussian Fits^a^

letter ID	*x* (cm^–1^)	*s* (cm^–1^)	*A*	E&P *x* (cm^–1^)
A	7385	13.5	72.4	
B	10187	19.6	1925	10185
C	10442	64	1528	10500
C	10526	54	4658	10500
D	10706	291	782	10700
E (not fit)				
F	12045	18.1	42204	12037
G	12643	85	932	12660
H	12866	49	27.1	b
I	15458	180	385	15420
J	16055	46.4	1199	16075
K	16880	143	252	b
L	17799	50	229	17800
M (not fit)	18570			
N	19111	299	4579	19100
O	19811	36	585	19810
P	21239	191	610	21200
Q	21828	55	410	21840

a Estimated errors x (line center) ± 5 cm-1, s (half width) ± 15%, Area (arbitrary units) ± 20%.

b Not assigned by E&P.

We fit the band of transitions in the 10,000–11,000 cm^–1^ range with five Gaussian lines which correspond to the four absorption features and the baseline fit for this energy region. In this region E&P also reported two peaks around 10500 cm^–1^.^9^ The corresponding region for the (NpO_2_)^1+^ spectrum^18^ showed only three absorption features. Given that two bands were separated by only ∼80 cm^–1^ and were relatively weak, we assigned only one transition in this region in accord with previous experimental and theoretical results. With these assignments for this region, our experimental energies agree for the most part with those given by E&P given the experimental uncertainties, with the addition of the assigned magnetic dipole transition at 7384 cm^–1^, Table 4.

### Initial Parameters and Fitting Procedure

We utilized the fitting programs developed by Prof. Michael Reid^34^ for calculations performed with the Russell–Saunders basis set. It is important to note that the magnitude of the axial crystal field for the actinyl ions is extremely large for an f^n^ ion. If we utilize the two data sets found by Denning et al. for the (NpO_2_)^2+^ ion diluted in single crystals of Cs_2_UO_2_Cl_4_ or CsUO_2_(NO_3_)_3_ we find the total crystal field splitting for this ion is between ∼42000 – ∼116,000 cm^–1^. In our analysis of the aqueous spectrum of the (NpO_2_)^+^ ion we found the total crystal field splitting to be ∼70,000 cm^–1^. The total splitting for the 5f^2^ free ion U^4+^ is approximately 50,000 cm^–1^ which includes the electrostatic repulsion between the two electrons and the effects of spin–orbit coupling.^35^ So for the actinyl 5f^2^ ions, the crystal field interaction is on the same order of magnitude or larger than the free ion interactions.

Numerous theoretical calculations have been carried out for the (NpO_2_)^1+^ and (PuO_2_)^2+^ ions using various levels of theory.The major difference between our data analysis In most cases these levels agree with the assignments of E&P.

As expected, the optical spectra for the actinyl ion (NpO_2_)^1+^ in the region from 0 to ∼11,000 cm^–1^ appears quite similar to that of the (PuO_2_)^2+^ except in the latter case there is a shift to higher energies (0–13,000 cm^–1^). For this reason, we expected the axial crystal field parameters to be rather similar. There are no detailed analyses of the optical spectra of plutonyl compounds so we started out by assuming that the crystal field parameters found for the neptunyl aqueous ion would be good initial guesses also for the plutonyl ion and that the ordering of the levels would be very similar to that found for the (NpO_2_)^+^ spectrum up to the most intense plutonyl level at 12,500 cm^–1^. For the initial free ion parameters, we utilized the plutonyl values for F^2^ and ζ_*f*_ as given by E&P and assumed the following ratios F^4^/F^2^ = 0.958, and F^6^/F^2^ = 0.626 as we had done previously in fitting the aqueous (NpO_2_)^1+^ spectrum. We then allowed all free ion parameters to vary. This procedure resulted in a reasonably good fit for the chosen levels although we had to substantially reduce the value of F^2^. We used these empirical parameters and calculated the energy of the second expected magnetic dipole transition as found for the isoelectronic (NpO_2_)^+^ ion. We assigned this transition and three other transitions based on their calculated energies and their correspondence with the (PuO_2_)^2+^ experimental levels.

It became clear that we had the same problem as found by Denning et al.^13^ that the value of the crystal field parameter σ was rather indeterminate. When fitting with the *B*_0_^*k*^ parameters this resulted in rather large errors in the parameter *B*_0_^6^. We determined the best way to perform the fitting procedure was to use all the levels that could be assigned by counting the doubly degenerate levels twice and to use fixed values of the parameter σ. By these methods we were able to get reasonable fits to the assigned data. We then fixed all parameters except σ and allowed that one parameter to fit. Subsequently we fixed σ at that value and resumed the fitting of all other parameters.

When using the *B*_0_^*k*^ fitting programs, we converted the E&P crystal field parameters to the *B*_0_^*k*^ crystal field parameters and fixed the value of *B*_0_^6^. The final results of these fitting procedures are shown in Table 5, with our best fit parameters given in Table 6 along with the parameters that were determined by E&P.

**Table 5:** Calculated and Experimental Energy Levels With Parameters from Table 6 That Gave the Best fit

level (D_∞h_)	calculated energy cm^–1^	exper. energy cm^–1^	wavefunct. (lz1 sz_1_, lz_2_ sz_2_) %(lz1 sz_1_, lz_2_ sz_2_) + %(lz1 sz_1_, lz_2_ sz_2_) two largest terms	wavefunct. (SLJJz) % (2S+1)L(J, Jz) + % (2S+1)L(J, Jz) Two largest terms	calc.^a^ f(10^–7^)	assign
E_4g_	0.8	0	91%(2– 3−) + 6%(2– 2+)	90% 3H(4 4) + 7% 3H(5 4)		GS
A_1g_	2143		46%(−2+ 2−) + 22% (−3+ 3−)	44% 3H(4 0) + 43% 3F(2 0)		
E_1g_	3823		56%(−3+ 2−) + 31% (−2– 2−)	37% 3F(2 1) + 29% 3H(4 1)		
E_5g_	7277	7385	56%(2+ 3−) + 43% (2– 3+)	91% 3H(5 5) + 9% 3H(6 5)	MD	A
E_1g_	10228	10187	48%(−2 + 2+) + 33% (−2+ 3−)	43% 3F(4 1) + 17% 3H(5 1)	103.8	B
A_1g_	10427	10500	33%(−2+ 2−) + 30% (−2– 3−)	47% 3F(4 0) + 20% 3F(2 0)		C
A2g	10593	10705	46%(−2– 3−) + 46% (−3 + 2+)	64% 3F(3 0) + 28% 3H(5 0)		D
E_2g_	12129	12045	86%(−3– 2−) + 8% (−2– 1−)	35% 3F(3 2) + 18% 3F(4 2)	1246	F
E_6g_	12943	12860	91%(2 + 3+) + 9%(3– 3+)	94% 3H(6 6) + 6% 1I(6 6)		H
E_4g_	13863		79%(2– 2+) + 10% (1 + 2+)	43% 3F(4 4) + 26% 1I(6 4)		
A_1g_	15684		55%(−3+ 3−) + 34% (−2– 2+)	36% 1D(2 0) + 18% 3P(0 0)		
E_3g_	16128	16055	85%(1– 3−) + 10% (1– −2+)	85% 3H(4 3) + 10% 3H(5 3)	MD	J
E_2g_	17836	17799	85%(1– 2−) + 7% (−1+ 3−)	41% 3H(4 2) + 32% 3F(2 2)	236.2	L
E_1g_	18260		66% (−2– 3+) + 20%(−3 + 3+)	32% 1D(2 1) + 22% 3P(1 1)		
A_1g_	19098	19111	35%(−2– 2+) + 19% (−3– 3+)	46% 1G(4 0) + 22% 3H(6 0)		N
E_5g_	19827	19811	46% (2– 3+) + 43% (2+ 3−)	85% 1I(6 5) + 12% 3H(6 5)	MD	O
E_2g_	20137		72% (−1+ 3−) + 10% (1– 2−)	33% 3F(4 2) + 28% 3F(2 2)		
E_1g_	21223	21260	60%(−1+ 2−) + 15% (−3 + 3+)	37% 3H(4 1) + 21% 3P(1 1)		P
E_4g_	21736	21828	55% 1+ 3−) + 29% (1– 3+)	71% 3H(5 4) + 10% 3H(6 4)	MD	Q
E_1g_	22556		83%(−1– 3−) + 7% (−1– 2+)	27% 3F(3 1) + 25% 3F(4 1)		

a Values are from Pitzer et al.^18^ calculated for the corresponding wave functions for the same state for the isoelectronic (NpO_2_)^1+^ ion.

**Table 6:** Parameters Obtained From Our Best fit (Col. 2) Compared With Values From E&P^a^

parameters	values	E&P parameters
cm^–1^	cm^–1^	cm^–1^
*E*_ave_	32117 ± 48	
F^2^	48182 ± 446	57195
F^4^	42364 ± 1589	44704
F^6^	19137 ± 1577	47210
ζ	2473.5 ± 13.5	2467
*B*_0_^2^	40692 ± 133	53457
*B*_0_^4^	58885 ± 248	74582
*B*_0_^6^	29480	44108
δ - φ	–1846	–1183
π - φ	13264	16793
σ - φ	37072	49650

a For Col.2–n, number of data points = 23, number of free parameters = 7, reduced rms deviation = 81.4 cm^–1^.

## Discussion

As shown in Table 5 the calculated levels up to approximately 16,000 cm^–1^ are primarily formed from the δ and φ orbitals so that the conventional crystal field theory we are using is applicable. Higher lying orbitals contain for the most part considerable contributions from the π orbitals as well as from δ and φ orbitals, which suggests that these calculated energies and wave functions are less reliable as conventional crystal field theory is not applicable for these types of orbitals under D_∞h_ symmetry.

When we compare our parameter list to that of E&P in Table 6, it is the F^k^ parameters show the most differences. The E&P parameter set has a very large F^2^ value and also F^6^ larger than F^4^. This is contrary to the definition of the F^k^ parameters as noted previously. The major difference between our data analysis and that of E&P is in the values obtained for the F^k^ parameters and that the F^2^ should be much smaller and closer to the F^2^ value found for the isoelectronic NpO_2_^+^ ion.

In Table 7 we show all the experimental parameters that have been reported for the 5f^1^ and 5f^2^ neptunyl and plutonyl species in the solid state and in solution. As noted earlier, the cf parameter σ is not well-defined, which results in the conventional crystal field parameters, *B*_0_^2^, *B*_0_^4^, and *B*_0_^6^ having a widespread of values. However, using the E&P crystal field parameters, there is a reasonable consistency across the series given the large uncertainties in these parameters. In addition, the free ion parameters going from the (NpO_2_)^1+^ ion to the (PuO_2_)^2+^ ion increase as one would expect from the increased nuclear charge on the plutonyl ionic species.

**Table 7:** Slater, Spin–Orbit and Crystal Field Parameters for Selected Actinyl Ions From Previous Studies and Present Results

parameters^a^	Cs_2_NpO_2_Cl_4_^a^ fit I (cm^–1^)	Cs_2_NpO_2_Cl_4_^a^ fit II (cm^–1^)	CsNpO_2_(NO_3_)_3_^a^ fit I (cm^–1^)	CsNpO_2_(NO_3_)_3_^a^ fit II (cm^–1^)	NpO_2_^+^^b^ (cm^–1^)	(PuO_2_^)2+^^c^ (cm^–1^)
ζ_*f*_	2196.1	2118.15	2212.0	2047.3	2069.1	2473.5
F^2^					46801	48182
F^4^					44823	42364
F^6^					29869	19137
*B*_0_^2^	95360.8	42099.3	96265	48050	62043	40692
*B*_0_^4^	157940.3	62823.2	158729	69318	98209	58885
*B*_0_^6^	173337.6	36891	170829	44405.6	94073	29480
ε_φ_	0	0	0	0	0	0
ε_δ_–ε_φ_	–1932.0	–1994.5	–2074.5	–1366.0	–1404	–1846
ε_π_–ε_φ_	13003.45	12626.0	13847.0	14179.8	11787	13264
ε_σ_–ε_φ_	114000.0	40000.0	114000.0	46000.0	69179	37072

a From ref (13) we are not including the off-diagonal crystal field parameters given by Denning et al. as they give only small shifts and split the degeneracy of some energy levels.

b From ref (19).

c Present results.

We can get some idea about how reliable our parameters are by using a reduced basis set in the < sz1lz1sz2lz2> basis and by excluding all matrix elements that contain sigma states, that is if any matrix element contains |0+> or |0→ we exclude that matrix element from our calculations. This results in a matrix of dimension 66 by 66.^7^ We can only carry out this calculation in this particular basis set. The results of this calculation (with restrictions on the values of F^4^ and F^6^ obtained from calculations with the full basis set) are shown at the bottom of Table 8. The values obtained are not too different than those found with the full 91 by 91 basis set. This finding suggests that the values found with the full basis set should be considered at least qualitatively accurate for the lowest levels of the configuration and clearly are the best that can be expected given the quality of the experimental data.

**Table 8:** Calculated Energy Levels with the Reduced Basis Set (No lz = 0 States)^a^

level (D_∞h_)	calculated energy cm^–1^	Exper. energy cm^–1^	Wavefunct. (lz1 sz_1_, lz_2_ sz_2_) %(lz1 sz_1_, lz_2_ sz_2_) + %(lz1 sz_1_, lz_2_ sz_2_) two largest terms
E_4g_	0	0	91%(2– 3−) + 6%(2– 2+)
A_1g_	2108		46%(−2+ 2−) + 22% (−3+ 3−)
E_1g_	3771		56%(−3+ 2−) + 31% (−2– 2−)
E_5g_	7280	7385	56%(2+ 3−) + 43% (2– 3+)
E_1g_	10211	10187	48%(−2 + 2+) + 33% (−2+ 3−)
A_1g_	10515	10500	33%(−2+ 2−) + 30% (−2– 3−)
A_2_g	10623	10705	46%(−2– 3−) + 46% (−3 + 2+)
E_2g_	12069	12045	86%(−3– 2−) + 8% (−2– 1−)
E_6g_	12912	12860	91%(2 + 3+) + 9%(3– 3+)
E_4g_	13863		79%(2– 2+) + 10% (1 + 2+)
A_1g_	15684		55%(−3+ 3−) + 34% (−2– 2+)
E_3g_	16128	16055^b^	85%(1– 3−) + 10% (1– −2+)
E_2g_	17836	17799^b^	85%(1– 2−) + 7% (−1+ 3−)
E_1g_	18260		66% (−2– 3+) + 20%(−3 + 3+)
A_1g_	19098	19111^b^	35%(−2– 2+) + 19% (−3– 3+)
E_5g_	19827	19811^b^	46% (2– 3+) + 43% (2+ 3−)
E_2g_	20137		72% (−1+ 3−) + 10% (1– 2−)
E_1g_	21223	21260^b^	60%(−1+ 2−) + 15% (−3 + 3+)
E_4g_	21736	21828^b^	55% 1+ 3−) + 29% (1– 3+)
E_1g_	22556		83%(−1– 3−) + 7% (−1– 2+)

a Final parameters for calculation with (sz1lz1sz2lz2) 66 × 66 basis set are as follows (all units in cm^–1^): zeta = 2468 ± 10, F^2^ = 47028 ± 873, F^4^ fixed at 0.879 * F^2^, F^6^ fixed at 0.397 * F^2^, ε_δ_–ε_φ_ = −1750 ± 221, ε_π_– ε_φ_ = 12578 ± 520. Reduced rms energy deviation 68 cm^–1^.

b Not used in the fitting the parameters below.

What is clearly needed is high quality low temperature experimental data on more (PuO_2_)^2+^ compounds. Early Russian work^14^ has shown that such data can be obtained but it is clear from their study, parts of which have been verified by later fluorescent experiments,^17^ that at higher energies, where the transitions are interrogating levels comprised of considerable amounts of π_u_ and σ_u_ orbitals, additional theory will be required to obtain a reliable fit to these data.

## Summary

We have refit the near-infrared and visible data for the solution absorption spectrum of the plutonyl ion in aqueous 1 M HClO_4_ assuming only axial symmetry. We note the full 91 by 91 Hamiltonian utilized for D_∞h_ symmetry is only strictly applicable to the δ and φ nonbonding orbitals of the 5f^2^ configuration. Our experimental energy level list is weighted toward the lower energy levels where the states consist primarily of the δ and φ orbitals, so the errors associated with this procedure are minimized. A calculation using a truncated basis set that excludes the antibonding σ orbitals resulted in Hamiltonian parameters not too different than those obtained with the full basis set. The results of our fitting procedures show substantial differences for the free ion F^k^ parameters from those found originally for the plutonyl ion in aqueous solution by Eisenstein and Pryce.^9^
